# Examining the Role of Spatial Changes in Bimodal and Uni-Modal To-Be-Ignored Stimuli and How They Affect Short-Term Memory Processes

**DOI:** 10.3389/fpsyg.2019.00299

**Published:** 2019-03-11

**Authors:** Erik Marsja, John E. Marsh, Patrik Hansson, Gregory Neely

**Affiliations:** ^1^ Department of Psychology, Umeå University, Umeå, Sweden; ^2^ School of Psychology, University of Central Lancashire, Preston, United Kingdom

**Keywords:** bimodal, short-term memory, vibration, audition, multisensory, distraction, attention capture, serial recall

## Abstract

This study examines the potential vulnerability of short-term memory processes to distraction by spatial changes within to-be-ignored bimodal, vibratory, and auditory stimuli. Participants were asked to recall sequences of serially presented digits or locations of dots while being exposed to to-be-ignored stimuli. On unexpected occasions, the bimodal to-be-ignored sequence, vibratory to-be-ignored sequence, or auditory to-be-ignored sequence changed their spatial origin from one side of the body (e.g., ear and arm, arm only, ear only) to the other. It was expected that the bimodal stimuli would make the spatial change more salient compared to that of the uni-modal stimuli and that this, in turn, would yield an increase in distraction of serial short-term memory in both the verbal and spatial domains. Our results support this assumption as a disruptive effect of the spatial deviant was only observed when presented within the bimodal to-be-ignored sequence: uni-modal to-be-ignored sequences, whether vibratory: or auditory, had no impact on either verbal or spatial short-term memory. Implications for models of attention capture and the potential special attention capturing role of bimodal stimuli are discussed.

## Introduction

That sudden and unexpected changes in a sequence of to-be-ignored (TBI) auditory stimuli can have a disruptive effect on cognitive performance is well known (e.g., see reviews by Hughes, 2014; Parmentier, 2014). Research has shown that these sudden and unexpected changes, known as deviants, have the behavioral consequences of prolonging responses in categorization tasks (e.g., Parmentier, 2014) and impairing memory for the order and identity of serially presented items in short-term memory tasks (e.g., serial-recall; Hughes et al., 2005, 2007). These effects are often referred to as attentional capture and have been reported in both uni-modal (e.g., task and TBI stimuli within the same modality; Berti, 2008) and cross-modal (e.g., task in the visual modality and TBI stimuli in the auditory modality; Hughes et al., 2005, 2007; Ljungberg and Parmentier, 2012) task settings. Cross-modal attentional capture is particularly interesting since the sensory environment is rarely based on stimulation in one modality at a time. Rather, experiences in daily life are based on information from many modalities simultaneously, or at least in close temporal alignment. For example, when waiting for the train at the train station, the ground below you may start vibrating at, more or less, the same time as when the sound of the incoming train arrives. Research investigating stimuli from two sensory modalities has shown that bimodal stimuli (e.g., stimuli from two sensory modalities) can capture attention and improve performance (e.g., the pip-and-poke effect in search tasks; Van der Burg et al., 2009).

In the present study, we aimed to address the question of whether the effects of bimodal distractors on cognitive performance are specific to particular sensory domains or whether the effects apply more generally to the perceptual system. Undertaking such study may prove fruitful for multisensory research in the sense that it would extend the research on bimodal stimuli to situations where it potentially affects short-term memory performance negatively (c.f. Santangelo et al., 2008).

There is a substantial literature on attentional capture by auditory deviants using different variants of the oddball paradigm. In this task, participants are exposed to a repetitive stream of the same stimulus (80% of trials). Another sudden and unexpected stimulus (deviant) is presented on rare occasions (e.g., 20% of the trials). It has been found that auditory (e.g., Berti and Schröger, 2003; Berti, 2008) visual (e.g., Czigler, 2007; Kimura et al., 2011; Stefanics et al., 2014), and tactile (Yamaguchi and Knight, 1991; Knight, 1996) deviant stimuli presented amidst a stream of repetitive stimuli in the same sensory domain capture attention. One important finding is that deviant, or novel, stimuli also disrupt performance in categorization tasks – response latencies are prolonged (see Parmentier, 2014 for an extensive review). Importantly, for the present study, it has further been reported that deviants also have a negative impact on short-term memory for serial order (e.g., serial-recall; Hughes et al., 2005, 2007; Lange, 2005). In the serial-recall task, participants typically encode to-be-recalled (TBR) items in the order that they appear while being exposed to TBI sequences. As in the oddball paradigm, the participants are exposed to the same sound in the majority of trials (e.g., 80%). On sudden and unexpected occasions (e.g., 20% of the trials), one of the sounds in the TBI sequence (e.g., the 5th in the sequence) is exchanged for a deviant sound. For example, a deviant could be a change in the temporal pattern of the repetitive stream of TBI sounds (Hughes et al., 2005), or a change from a male to a female voice (Hughes et al., 2007; Sörqvist, 2010). Generally, performance drops of up to 10% have been observed in serial recall tasks wherein auditory deviants were presented (Marsh et al., 2014; Röer et al., 2014a,b).

As far as we know, there are no studies examining whether deviations in vibrating and bimodal (e.g., auditory and vibratory) TBI sequences affect STM performance. The majority of previous research has focused on how attention can be captured to spatial locations by the presentation of tactile or bimodal cues. For example, the presentation of a tactile cue prior to a target decreases response latencies for targets in the same location (e.g., Santangelo et al., 2008). In visual search tasks, it has been found that audiotactile cues can lead pre-attentively and automatically to multisensory integration in a bottom-up fashion, which then makes it more probable that the resulting event will capture attention and thus seize available processing resources (Van der Burg et al., 2009). Bimodal cues have also been found to efficiently capture attention even though there is a high perceptual load (e.g., audiotactile cues; Santangelo et al., 2008; audiovisual cues; Santangelo and Spence, 2007). Deviant vibrations have been shown to capture attention from categorization tasks (Parmentier et al., 2011c): an effect that has been found to be functionally similar to attention capture by deviant stimuli (Ljungberg and Parmentier, 2012). Finally, it has also been reported that an omission of a standard vibration can capture attention (Marsja et al., 2018). Although bimodal deviants have been reported to capture attention using the oddball paradigm, the effect has not been found to be larger than auditory deviants (Boll and Berti, 2009).

Concerning STM, Botta et al. (2011) examined the effect of visual, auditory, and audiovisual cues on STM performance using a change detection task. They used both cross-modal and modality-specific cues that could either be congruent or incongruent with the spatial location of upcoming to-be-recalled (TBR) items. In the congruent condition, attention was captured towards the spatial location that contained the TBR items, whereas in the incongruent condition, attention was captured toward the opposite spatial location to the TBR items. It was found that audiovisual cues influenced performance accuracy to a larger extent than uni-modal cues (visual or auditory). Botta et al. (2011) found that response accuracy increased when the audiovisual cue was congruent and decreased when the audiovisual cue was incongruent. Crucially, both congruent and incongruent cues had a larger effect when they were bimodal compared to visual cues only.

A typical explanation of the results observed by Botta et al. (2011) is that of multisensory integration. Multisensory integration is the set of processes that enable sensory information (e.g., auditory, visual, tactile) to interact and affect processing in other sensory modalities. Importantly, this includes how sensory information can be combined to create one percept (e.g., Talsma et al., 2010). In the context of the foregoing studies, it has typically been argued that this unified percept creates an increased perceptual saliency compared to uni-modal stimuli (e.g., Santangelo and Spence, 2007; Santangelo et al., 2008; Botta et al., 2011).

In the present study, we changed the location of the TBI stimuli from one side of the body to the other. We assume that this change in the location of the TBI stimuli (a spatial deviation) will be more salient in the context of a bimodal TBI sequence than a uni-modal TBI sequence. For example, if the TBI sequence is auditory, the sound will move from being presented in the left ear to the right ear, and when the TBI sequence is vibrotactile uni-modal, the vibration will move from the left arm to the right arm. Using a spatial deviant in this manner yields the advantage of making it possible to compare effects between modalities without having to change the physical characteristics of the stimuli. Typically, deviants in sensory stimuli have involved a change in a physical component of the stimulus, such as frequency level in sound or intensity of a vibration. However, when studying bimodal sensory environments, it often difficult to find manipulations that would create deviant stimuli in both modalities that the respondent would judge to be equivalent changes from the standard. Thus, it is often difficult when comparing results between the modalities to know if the differential effects are due to the presence of a deviant per se or due to the fact that, e.g., a change in the frequency of a sound is more distracting than the change of intensity of a vibration. We hypothesize that bimodal TBI sequences with spatial deviants will have larger negative impacts on STM performance than the same changes in uni-modal TBI sequences.

## Methods and Materials

### Participants

One hundred and fifty students at Umeå University took part in the study and were divided into three TBI stimuli exposure groups (see Table 1). Participants received a small honorarium of approximately $12 for taking part in the study. All participants reported normal or corrected-to-normal vision and hearing as well as no somatosensory deficits. The study reported here has been carried out in accordance with the Declaration of Helsinki. This study was deemed exempt for ethical review by the Ethics Committee at Umeå University as the stimuli were harmless and no personal information was to be collected (DNR 2012-337-31Ö). Before the experimental session started, the participants gave written informed consent.

**Table 1:** Sample description in terms of biographical variables.

TBI	*N*	Male	Female	Other	Age range	Mean age (*SD*)
Bimodal	50 [45]	16 [14]	34 [31]	0 [0]	18–42 [18–42]	27.2 (5.55) [27.3 (5.57)]
Vibrotactile	50 [47]	21 [20]	28 [26]	1 [1]	18–37 [18–37]	25.78 (4.51) [25.9 (4.54)]
Auditory	50 [44]	21 [19]	28 [25]	1 [0]	18–39 [18–39]	27.12 (5.18) [27.2 (4.95)]

Participants included in analysis within brackets.

### Apparatus and Materials

The experiment was programmed using Python and PsychoPy (Peirce, 2007) and was executed on computers running Windows 7 Enterprise Edition. The to-be-remembered (TBR) visual stimuli were presented on 24-inch widescreen LCD-monitors. The TBI vibrotactile stimuli were comprised of 10 repetitions of a vibration of 240 Hz, and the amplitude of 1.8 g (peak-to-peak), or 8.0 μm. Vibrotactile stimuli were delivered using two brushless coin vibration motors (Dura Vibe model 910–101, 10 mm, 3 V, 65 mA, 12,500 rpm, 1 g; Precision Microdrives). The motor’s surface area (point of contact) was 74.5 mm^2^ and reached maximal rotation speed after approximately 52 ms. Each motor was attached to the upper arms using elastic webbing. The vibration motors were controlled by an Arduino Uno board rev. 3 and programmed using the Arduino IDE, version 1.6.6 (www.arduino.cc).

The TBI auditory sequences were comprised of 10 repetitions of a 600 Hz sinewave tone. The auditory sequences were created using Audacity 2.1.2 and were presented binaurally through Vic Firth sound attenuated headphones. Each stimulus in the TBI sequences had an inter-stimulus-interval (ISI) of 250 ms. Both the vibrotactile and auditory stimuli had durations of 250 ms (auditory stimuli had 10 ms on and off ramps) and were presented simultaneously (henceforth, referred to as bimodal TBI sequence). All participants, across all TBI exposure groups, wore the sound attenuated headphones to mask the sound of the vibrating motors.

#### Serial Recall Tasks

A verbal and spatial serial-recall task was used (e.g., Lange, 2005; Vachon et al., 2017). Both the tasks and the TBI sequence were designed to follow the methodology of Vachon et al. (2017). That is, the same amount of TBR items and TBI items were used, and the position of the deviant in the TBI sequence was the same.

The TBR lists in both the verbal and spatial tasks consisted of seven items. In the verbal task, the seven items were taken randomly without replacement from the digit set 1–9. Each digit was presented in Arial font at the center of the screen. In the spatial task, the seven items were taken randomly without replacement from a 5 × 5 matrix. Each dot was 1 cm in diameter. All items, whether verbal or spatial, were presented in black on a white background. The ISI in the TBI sequence was 350 ms, whereas the ISI between TBR items in the relevant sequences, in both tasks, was 450 ms. These timings were adopted to prevent any systematic synchronicity between the relevant and TBI items.

### Procedure

An experimental session started with general instructions concerning the serial recall task. After that, two practice trials of each of the tasks were given. Instructions on the specific task (verbal or spatial) were given prior to the two practice trials. The participants were told to recall the order of seven digits in the verbal practice trials and were instructed to recall the locations of the seven dots in the spatial practice trials. Participants were informed that all other sensory stimuli were irrelevant to the task and were to be ignored.

**Figure 1:**
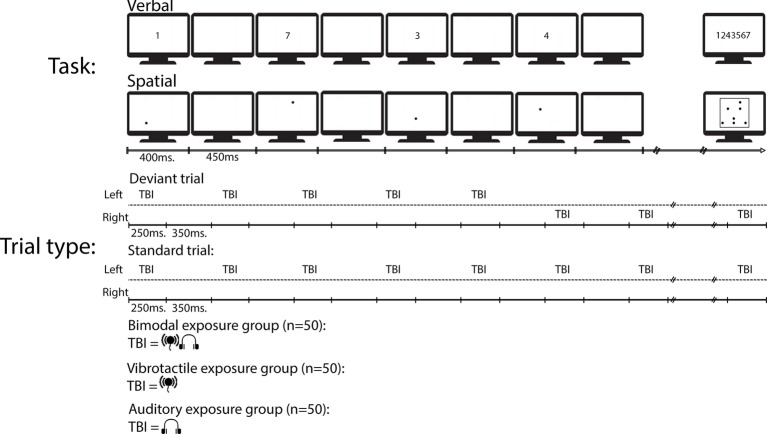
A schematic view of an example deviant and an example standard trial type in the experiment. To-be-ignored (TBI) stimuli were either presented on the same time of the body during an entire trial (i.e., standard) or changed side of the body during deviant trial types.

In 80% of the trials, the TBI sequence was presented on the same side of the body, and in roughly 21% of the trials (15 trials per task; henceforth called spatial deviant), the TBI sequence changed side at the 6th stimulus in the TBI sequence (e.g., from left to right) and continued being presented at the new side until the next deviant caused a new change of side. The TBI sequences differed between each group such that there was one exposure group with bimodal TBI sequences, one exposure group with only vibrotactile TBI sequences, and one group with only auditory TBI sequences (see Figure 1 for a schematic overview). The tasks were blocked and there were three blocks, 24 trials in each block, of both tasks (in total six blocks). The order of the tasks was counterbalanced across the participants. Each block was started by pressing the space bar.

To start a trial, the participants had to click the mouse inside a rectangle containing the text “start trial.” The rectangle remained on the screen for 1,000 ms before the first of seven digits, or seven dots, were presented. On the response screen of both tasks, participants were required to click the serial order of the digits or dots, once all of the TBR items appeared on the screen. In the verbal task, the digits were presented across the middle of the screen in canonical order. Each item turned green once selected, and omissions were not allowed. Once participants had given their answer, the start trial rectangle reappeared. See Figure 1 for a schematic view over a typical trial. Including optional breaks, the experiment lasted approx. 50 min.

### Data Analysis

Mean proportion of correct recalled items was calculated for each trial type (i.e., standard and deviant) with a strict criterion (right spot). All data were processed and analyzed within the R statistical programming environment (R Core Team, 2017). To allow for visual comparisons between conditions the 95% confidence intervals of the means were computed according to Cousineau (2005) and Morey (2008). A mixed ANOVA with the between-subjects factor TBI sequence (bimodal, vibrotactile, and auditory) and within-participant trial type (deviant and standard) was conducted with type-III sum of squares using the function *aov_ez* in the r-package afex (Singmann et al., 2018). Planned comparisons were computed using the functions *emmeans* and *contrast* in the package emmeans (Lenth, 2019). The confidence intervals for the pairwise comparisons were also computed using emmeans and are reflecting the difference between conditions.

## Results

Due to hardware failure and poor performance (e.g., performance under 30% correct in one of the two tasks), five participants from the bimodal exposure group and three participants from the vibrotactile exposure group were excluded from the analysis. In the auditory exposure group, six participants performed poorly and were thus excluded from the analysis. Thus, in the following analysis, data from 45 (bimodal exposure group), 47 (vibrotactile exposure group), and 44 (auditory exposure group) were analyzed.

**Figure 2:**
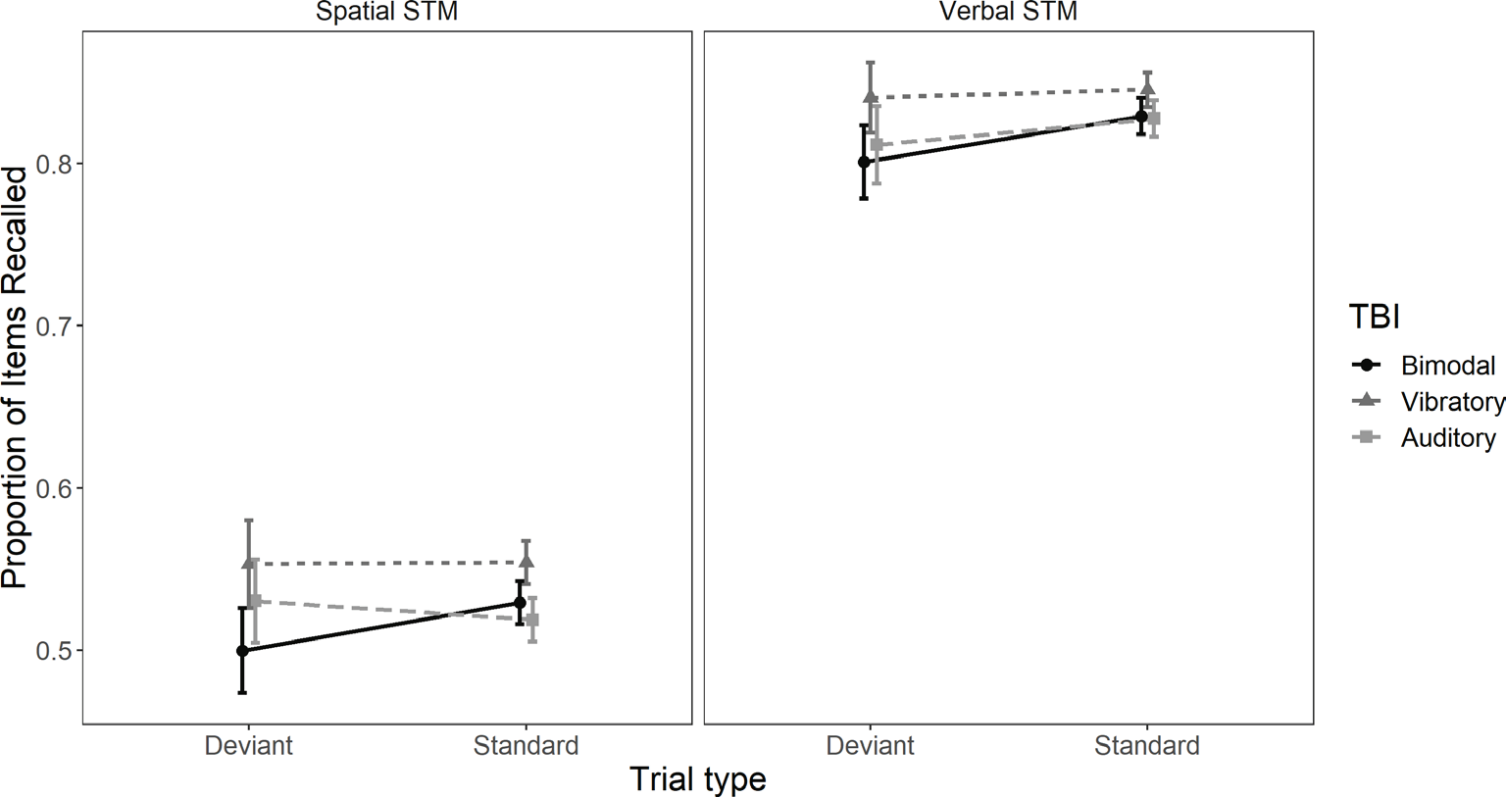
Mean proportion of items correctly recalled in trials with and without a spatial deviant. The left panel is depicting proportion of correctly recalled items in the spatial task and the right panel proportion of correctly recalled items in the verbal task. Error bars represent 95% confidence intervals.

There was no main effect of TBI exposure group, *F*(2, 133) = 1.12, *MSE* = 6.85, *p* = 0.33, $\eta_{p}^{2}$ = 0.02, indicating that performance was not affected by bimodal and uni-modal TBI sequences. However, there was an effect of task, *F*(1, 133) = 462.026, MSE = 3.43, *p* < 0.01, $\eta_{p}^{2}$ = 0.78 and trial type *F*(1, 133) = 7.1, *MSE* = 0.326, p = 0.009, $\eta_{p}^{2}$ = 0.05. Crucially, there was a TBI sequence × trial type interaction, *F*(2, 133) = 3.84, *MSE* = 0.326, *p* = 0.024, $\eta_{p}^{2}$ = 0.05. The TBI × task interaction was neither significant, *F*(2, 133) = 0.04, *MSE* = 0.002, *p* = 0.95, $\eta_{p}^{2}$ = 0.0006, nor was the task × trial type interaction, *F*(1, 133) = 0.65, *MSE* = 0.002, *p* = 0.42, $\eta_{p}^{2}$ = 0.005, nor the TBI × task × trial type interaction, *F*(2, 133) = 0.16, *MSE* = 0.002, *p* = 0.85, $\eta_{p}^{2}$ = 0.002. These results indicate that the spatial deviant had a negative effect on performance in both verbal and spatial STM tasks. See Figure 2 for performance across trial types and tasks.

Planned comparisons revealed that the effect of trial type was different in the bimodal TBI sequence compared to the vibrotactile TBI sequence, *t*(133) = −2.46, *p* = 0.015, 95% *CI* [−0.046, −0.005] and the bimodal TBI sequence compared to the auditory TBI sequence, *t*(133) = −2.34, *p* = 0.021, 95% *CI* [−0.045, −0.004]. However, the effect of trial type was statistically comparable between the vibrotactile TBI sequence and the auditory sequence, *t*(133) = 0.08, *p* = 0.94, 95% *CI* [−0.019, 0.02].^1^

## General Discussion

The goal of the current study was to examine whether a change of location in a bimodal or uni-modal TBI sequence affects spatial and verbal STM performance. This was achieved using spatial and verbal serial-recall tasks that were completed in the presence of vibratory and auditory TBI sequences. On unexpected occasions, the location of the TBI sequence changed from one side of the body to the other (i.e., spatial deviant). We found spatial changes in bimodal (i.e., both auditory and vibratory) TBI sequences disrupted performance in both spatial and verbal short-term memory tasks. However, no significant effect was found when the same spatial change was employed in uni-modal TBI sequences, either vibratory or auditory.

Attentional capture by deviant sounds is not contingent upon tasks taxing STM (e.g., Hughes et al., 2005, 2007; Lange, 2005) and has been shown in cross-modal task settings using auditory (e.g., Parmentier, 2014) and vibratory (Parmentier et al., 2011b; Ljungberg and Parmentier, 2012; Marsja et al., 2018) TBI stimuli. Furthermore, deviant stimuli have also been found to elicit three brain responses. The first two are of particular interest for the present study since they have been found using auditory, tactile, and visual stimuli. First, there is the mismatch negativity (MMN) which has been suggested to be an indication of a change-detection mechanism (e.g., Näätänen et al., 2007) or a marker for a violation of predictions (e.g., Bendixen et al., 2012; Winkler and Czigler, 2012). Second, the P3a is elicited when attention has been oriented to the change (i.e., the deviant stimulus; e.g., Friedman et al., 2001; Berti, 2008). Third, when participants are performing a primary task, such as judging the parity of visual digits, the re-orienting negativity (RON) is elicited. RON has been suggested to be a marker that attention has been re-oriented to the visual task (e.g., Schröger and Wolff, 1998; Berti, 2008). These brain responses have further been found to be elicited even when attention is focused on another modality (e.g., the visual modality when the to-be-ignored modality is auditory; Schröger and Wolff, 1998).

The results from our study are comparable with the study by Vachon et al. (2017) in which both verbal and spatial deviants affected STM. More specifically, the results from the present study add to the study by Vachon et al. (2017) by showing that STM is disrupted by an unexpected change that is both spatial and verbal. Furthermore, in a study by Morey and Miron (2016), auditory deviants were found to affect both spatial and verbal STM. In their study, verbal (i.e., spoken letters) and spatial serial-recall tasks were used. Of particular relevance for the present study, was that on infrequent trials in Morey and Miron’s study one of the TBR items changed character (i.e., deviant). In the verbal task, the deviant was a change from a female to a male voice, and in the spatial task, the deviant was a change of color. Since the tasks were either presented simultaneously or alone, Morey and Miron (2016) were able to explore the effects of both visual and auditory deviants in both tasks. They found that the auditory deviant disrupted performance in both tasks.

However, in the current study, presenting a spatial deviant within a vibratory or auditory TBI sequence failed to capture attention and impair STM performance. Differences in terms of the methodology adopted in our study relative to that of Vachon et al. (2017) could, of course, contribute to the apparent discrepancies in results. In their study, the TBI sequence was comprised of spoken letters (e.g., “A”) repeatedly presented, and the spatial deviant was a momentary change from one side for one stimulus and then back to the original side. In our study, we used sinewave tones and the TBI sequence changed side (i.e., spatial deviant) and continued on that side until the next deviant trial.

One could argue that letters, and changing the location of the letters, are more salient compared to a sinewave tone. For instance, even though letters carry very little semantic information, they have a meaning. Sinewave tones, on the other hand, carry less information. In the current study, we aimed to strip away as much semantic information as possible to be able to also compare auditory and tactile TBI stimuli, and it may be this aspect which underlies the difference in results between our study and that of Vachon et al. (2017). There are other methodological differences between the current study and the study Vachon et al. (2017). In the current study, the spatial deviant continued being presented at the side it changed to. In the study by Vachon and colleagues, on the other hand, after the spatial deviant was presented (e.g., the letter “A” was presented in the left ear instead of in the right ear), the following sounds in the sequence were presented in the same ear as prior to the deviant (e.g., in right ear). This could, in turn, have caused the spatial deviant to become a “double deviant.” Tentatively, this could also have made the spatial deviant in the auditory TBI sequence more salient in the study of Vachon et al. (2017). As far as we know, no study has previously examined how changes in location (i.e., a spatial deviant) using sinewave tones affect STM performance. Thus, on the basis of our results, it is hard to draw firm conclusions regarding whether uni-modal spatial deviants, whether auditory or tactile, are incapable of capturing attention. Furthermore, the overall performance in the current study was worse in the spatial task compared to the study of Vachon et al. (2017). Tentatively, this may be due to the fact that the TBI sequences in the current study were more distracting to the performance in the spatial task. Future research should employ a “no stimulation” condition to shed light on this. Such a study may also be able to further explore the differences between bimodal and uni-modal TBI sequences.

Our results do, however, suggest that there is a special role for spatial deviants when presented in bimodal TBI sequences. This is in line with data from studies showing a special role for bimodal (audiotactile) cues. For instance, bimodal spatial cues have been reported to be more effective distractors compared to uni-modal cues during high perceptual load (Santangelo et al., 2008; Ho et al., 2009). For example, in the study by Ho et al. (2009), participants were to respond to the elevation of peripheral visual targets. Prior to each visual target, an auditory cue was presented at the same side as the visual cue. Furthermore, the auditory cue was presented either alone or at the same time as a tactile cue. Interestingly, the auditory cue failed to capture attention toward the visual target when the participants were engaged in a perceptually demanding rapid serial visual presentation task. According to the perceptual load theory (Lavie, 2005), participant’s perceptual resources are necessarily and unavoidably used to process stimuli until the resources have run out. Based on this hypothesis, Santangelo et al. (2008) assessed attentional capture effects following auditory, tactile, and audiotactile (bimodal) exogenous cues under conditions of no load and high perceptual load. They found that attention was captured by both uni- and bimodal cues under low perceptual load. However, only the bimodal cue captured attention in the high load condition. This indicates that multisensory integration might be unique in disengaging spatial attention from a simultaneous perceptually demanding task (see also Santangelo and Spence, 2007). Santangelo et al. (2008) suggested that the presentation of bimodal stimuli (audiotactile) increased the perceptual saliency of the cues. Furthermore, existing research has also shown that bimodal cues increase performance in spatial STM task (Botta et al., 2011). In the study by Botta et al. (2011), the task was to remember colored rectangles presented in an array. Prior to each array, either a visual, an auditory, or an audiovisual cue was presented. Following a short retention interval, participants were required to decide whether a specific rectangle (marked with a surrounding rectangle) was the same color as the rectangle presented in the memory array. The cue could be either spatially informative or uninformative (i.e., indicating which side the to-be-remembered item was going to be presented). Botta et al. (2011) found that the bimodal cue improved the performance, whereas the uni-modal cues did not and suggested that this was due to the fact that the cues were multisensory integrated (see also Mastroberardino et al., 2008 for an extensive review on the advantage of bimodal information on short-term memory).

Similarly, we suggest that our results are due to the sounds and vibrations being integrated into one unitary sequence. This percept, in turn, could be more salient compared to the uni-modal sequences (i.e., auditory or vibratory), thereby rendering the spatial deviant more salient. This could explain why only the spatial change in the bimodal TBI sequence successfully captured attention. Intriguingly, temporally and spatially congruent stimuli from different modalities have been shown to have a higher likelihood of being further processed and thus to capture attention, compared to stimuli that are not temporally and spatially congruent (e.g., speed up responses in visual search tasks; Van der Burg et al., 2008, 2009; Ngo and Spence, 2010). It may worth noting, however, that introducing a spatial change in one of the modalities in the bimodal TBI sequence may very well capture attention away from the task. One recent explanation for attention capture by deviant stimuli posits that deviants capture attention because they violate the cognitive system’s prediction of upcoming stimulation (Parmentier et al., 2011a; Nöstl et al., 2012). For instance, if the auditory stream changes side, from left to right, while the tactile modality continues on the same side, it could be perceived as a violation of prediction. Future research should examine the effects of changes in one of the modalities within the bimodal TBI sequence.

In conclusion, spatial deviants in the bimodal TBI sequence influenced performance negatively in both verbal and spatial memory tasks. This may be due to the fact that the bimodal TBI sequence was more salient and thus made the spatial change harder to ignore. Our experiences in daily life are often based on information coming from multiple sensory pathways; e.g., when waiting for the train, the ground below may start vibrating at in close temporal alignment as when the sound of the incoming train arrives. Here, we empirically demonstrate this phenomenon (i.e., multisensory integration) using distraction as a vehicle.

## Data Availability

The datasets generated for this study are available on request to the corresponding author.

## Author Contributions

EM, JM, PH, and GN developed the research questions and wrote the Introduction, Materials and Methods, and Discussion sections. EM carried out the data analysis and, thus, wrote the Results section and created tables and figures.

### Conflict of Interest Statement

The authors declare that the research was conducted in the absence of any commercial or financial relationships that could be construed as a potential conflict of interest.

## Footnotes

^1^We also carried out the same analysis for the first five TBR items, and the results follow the pattern of the analysis of when analyzing all TBR items. That is, there was no main effect of TBI, *F*(2, 133) = 0.50, *MSE* = 0.05, *p* = 0.61 .33, $\eta_{p}^{2}$ = 0.008. However, there was an effect of task, *F*(1, 133) = 496.50, MSE = 0.03, *p* < 0.01, $\eta_{p}^{2}$ = 0.79 and trial type *F*(1, 133) = 4.70, *MSE* = 0.0, *p* = 0.03, $\eta_{p}^{2}$ = 0.03. Importantly, there was a TBI sequence × trial type interaction, *F*(2, 133) = 3.16, *MSE* = 0.0, *p* = 0.05, $\eta_{p}^{2}$ = 0.05. The TBI × task interaction was not significant, *F*(2, 133) = 0.09, *MSE* = 0.03, *p* = 0.92, $\eta_{p}^{2}$ = 0.001, nor was the task × trial type interaction, *F*(1, 133) = 0.16, *MSE* = 0.00, *p* = 0.69, $\eta_{p}^{2}$ = 0.001, nor the TBI × task × trial type interaction, *F*(2, 133) = 0.56, *MSE* = 0.00, *p* = 0.57, $\eta_{p}^{2}$ = 0.008. Furthermore, the same pattern appeared for the planned comparisons, it revealed that the effect of trial type was different in the bimodal TBI sequence vs. in the vibrotactile TBI sequence, *t*(133) = −1.983, *p* = 0.0494, 95% *CI* [−0.041, −0.00005] and the bimodal TBI sequence vs. in the auditory TBI sequence, *t*(133) = −2.34, *p* = 0.0212, 95% *CI* [−0.00437, −0.045]. Finally, the effect of trial type was still statistically comparable between the vibrotactile TBI sequence and the auditory sequence, *t*(133) = 0.39, *p* = 0.79, 95% *CI* [−0.0166, 0.025]. These results show that errors are increased for items presented even before the deviant onset. This back propagation of errors has been shown previously (Hughes et al., 2005) and indicates that the deviant affects memory per se, rather than just encoding.
